# Pain Neuroscience Education Plus Usual Care Is More Effective than Usual Care Alone to Improve Self-Efficacy Beliefs in People with Chronic Musculoskeletal Pain: A Non-Randomized Controlled Trial

**DOI:** 10.3390/jcm9072195

**Published:** 2020-07-11

**Authors:** Antonio Rondon-Ramos, Javier Martinez-Calderon, Juan Luis Diaz-Cerrillo, Francisco Rivas-Ruiz, Gina Rocio Ariza-Hurtado, Susana Clavero-Cano, Alejandro Luque-Suarez

**Affiliations:** 1 Servicio Andaluz de Salud, Distrito de Atención Primaria Costa del Sol, U.G.C. Las Lagunas, 29650 Mijas, Málaga, Spain; antonio.rondon.sspa@juntadeandalucia.es; 2 Universidad de Málaga, Facultad de Ciencias de la Salud, Departamento de Fisioterapia, 29071 Málaga, Spain; javier_martinez_calderon@hotmail.com; 3 Instituto de Investigación Biomédica de Málaga (IBIMA), 29010 Málaga, Málaga, Spain; 4 Servicio Andaluz de Salud, Distrito de Atención Primaria Costa del Sol, U.G.C. La Carihuela, 29620 Torremolinos, Málaga, Spain; juanl.diaz.cerrillo.sspa@juntadeandalucia.es; 5 Research Unit, Agencia Sanitaria Costa del Sol, 29603 Marbella, Málaga, Spain; frivasr@hcs.es; 6 Red de Investigación en Servicios de Salud en Enfermedades Crónicas (REDISSEC), 29603 Marbella, Málaga, Spain; 7 Servicio Andaluz de Salud, Distrito de Atención Primaria Costa del Sol, U.G.C. San Pedro de Alcántara, 29670 Marbella, Málaga, Spain; ginar.ariza.hurtado.sspa@juntadeandalucia.es; 8 Servicio Andaluz de Salud, Distrito de Atención Primaria Costa del Sol, U.G.C. Las Albarizas, 29600 Marbella, Málaga, Spain; susana.clavero.sspa@juntadeandalucia.es

**Keywords:** chronic pain, self-efficacy, musculoskeletal pain, education

## Abstract

Self-efficacy beliefs are associated with less physical impairment and pain intensity in people with chronic pain. Interventions that build self-efficacy beliefs may foster behavioral changes among this population. A non-randomized trial has been carried out to evaluate the effectiveness of pain neuroscience education (PNE) plus usual care in modifying self-efficacy beliefs, pain intensity, pain interference and analgesics consumption in people with chronic musculoskeletal pain. Participants were allocated to an experimental (PNE plus usual care, *n* = 49) and a control (usual care alone, *n* = 51) group. The primary outcome was self-efficacy beliefs (Chronic Pain Self-Efficacy Scale), and the secondary outcomes were pain intensity, pain interference (Graded Chronic Pain Scale) and analgesics consumption. The participant’s pain knowledge (revised Neurophysiology of Pain Questionnaire) after PNE intervention was also assessed to analyze its influence on every outcome measure. All the outcome measures were assessed at the baseline and at four-week and four-month follow-ups. PNE plus usual care was more effective than usual care alone to increase self-efficacy beliefs and decrease pain intensity and pain interference at all follow-up points. No differences between groups were found in terms of analgesics consumption. Knowledge of pain neurophysiology did not modify the effects of PNE plus usual care in any of the outcome measures. These results should be taken with caution because of the non-randomized nature of this design, the limited follow-ups and the uncertainty of the presence of clinical changes in self-efficacy for participants. Larger, methodological sound trials are needed.

## 1. Introduction

A positive attitude is probably the first step needed to engage individuals with chronic musculoskeletal (MSK) pain in self-management strategies [1]. In this context, self-efficacy beliefs refer to the belief that a person can manage a determined functional and/or emotional state and execute certain actions/activities despite the presence of a chronic disorder [2]. Self-efficacy beliefs foster the use of positive adherence behaviors [3] and are associated with less depressive symptoms, physical impairment, disease activity, emotional distress, fatigue and pain intensity in people with chronic pain [4,5]. Self-efficacy beliefs are also considered a mediator in the association between pain and depression [6], pain and disability [7], as well as pain and engagement in valued activities [8]. Moreover, self-efficacy beliefs moderate the effects achieved after applying behavioral changes and interventions such as cognitive–behavioral therapy [9].

Self-efficacy beliefs promote the attainment of valued goals such as regular exercise, which is essential as exercise is considered the first line of treatment in chronic MSK pain [10,11]. Therefore, interventions that build self-efficacy beliefs may foster behavioral changes among individuals with chronic pain, such as greater physical activity participation and adherence to positive sleep habits. This may improve the quality of life in this population [12]. Mastery and vicarious experience, verbal persuasion and education about the body’s response should be considered when pain clinicians aim to enhance self-efficacy beliefs [13]. Multicomponent, psychological and exercise interventions are promising therapeutic options to improve self-efficacy (specifically pain self-efficacy) in people with chronic MSK pain [14]. Additionally, pain education strategies have shown good results in improving self-efficacy beliefs when pain education is incorporated into a multicomponent program [14,15]. However, no trials have evaluated the effectiveness of adding pain education to usual care to modify self-efficacy beliefs in people with chronic pain. Pain neuroscience education (PNE) is an effective intervention to increase knowledge about pain neurophysiology among individuals with chronic pain [16], health professionals [17] and health students—e.g., physiotherapists—[18], and it is also effective for reconceptualizing pain perceptions, which is vital in order to decrease uncertainties about the chronic pain process. This intervention has also been found to produce low clinical relevance effects in decreasing pain and disability, but medium clinical relevance effects in reducing psychological factors such as kinesiophobia and pain catastrophizing in people with chronic MSK pain [19].

Given all these considerations, the following non-randomized controlled trial was aimed at evaluating whether the application of PNE plus usual care was more effective than usual care alone to improve self-efficacy beliefs in people with chronic MSK pain. This study also tested whether PNE plus usual care was more effective than usual care alone to reduce pain intensity, pain interference and analgesics consumption. Finally, in a secondary analysis, we evaluated whether the knowledge about pain neurophysiology acquired by participants in the intervention group (PNE plus usual care) after treatment did/did not influence the effects of PNE in any of the outcome measures. We hypothesized that PNE plus usual care would be more effective than usual care alone to enhance self-efficacy beliefs and decrease pain intensity, pain interference and analgesics consumption. We also hypothesized that a greater knowledge in pain neurophysiology would increase the effects of PNE plus usual care on all the outcome measures mentioned above.

## 2. Materials and Methods

### 2.1. Study Design

A pragmatical, non-randomized controlled trial was conducted, following the Declaration of Helsinki, the Guidelines for Reporting Non-Randomized Studies [20] and the Template for Intervention Description and Replication (TIDieR) checklist [21]. This study was approved by the Costa del Sol Ethics of Research Committee in January 2018 (code: 004_ene18_MR1-Educacion Neurociencia) and was registered in ClinicalTrials.gov (NCT03100721). A non-randomized design was chosen as the professionals who administer pain neuroscience education (PNE) (intervention group) require specific training and skills. Its use is not very widespread in the healthcare district where this study was conducted. Thus, PNE plus usual care intervention was administered in two centers, while usual care was administered in the other two.

### 2.2. Participants and Setting

This study was conducted in four physiotherapy primary care units of Malaga, Spain. Pain neuroscience education plus usual care was delivered in two units, while usual care alone was delivered in the rest of the units. A total of four physiotherapists recruited participants between April 2018 and January 2020. Participants who satisfied the eligibility criteria were invited to participate in this study and signed a written informed consent form. Physiotherapists also delivered an information sheet to all the participants with details about the study process. The inclusion criteria were as follows: aged 18–65 years; with chronic MSK pain, according to the ACTTION–American Pain Society Pain Taxonomy (AAPT) for chronic pain [22]. Participants with chronic axial MSK lower back pain were also included [22].

The exclusion criteria were as follows: chronic postoperative MSK pain and chronic MSK pain related to a traumatic injury six months before the beginning of the trial; inability to provide written informed consent and/or complete questionnaires in Spanish.

### 2.3. Allocation

**Figure 1:**
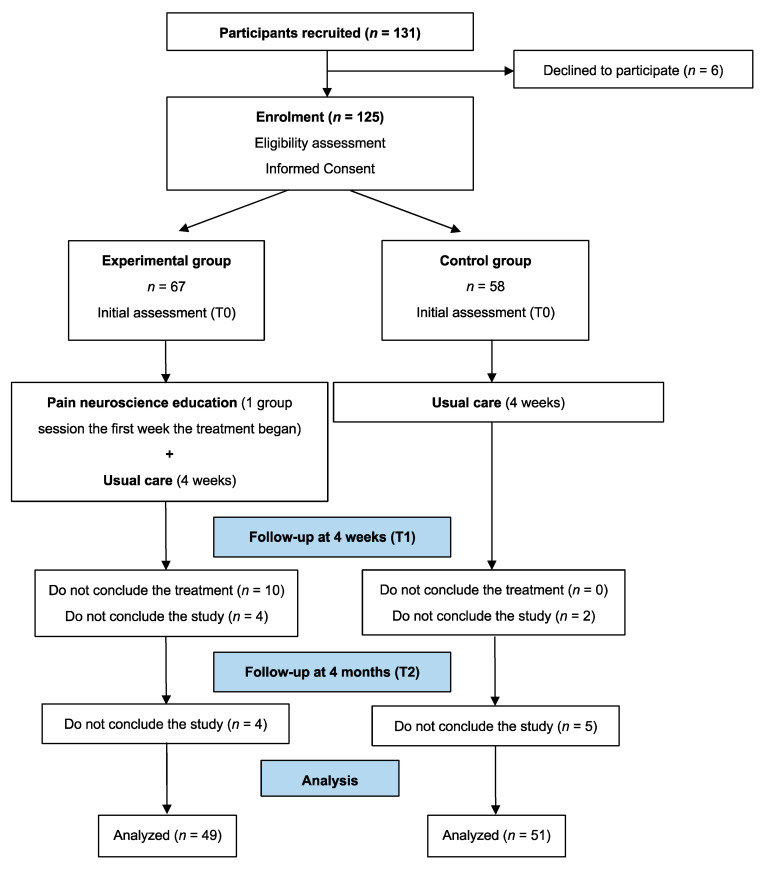
Flow diagram of the study.

The physiotherapists who led each group recruited and allocated the participants. Participants were allocated to the experimental group in two units, while participants were allocated to the control group in the rest of the units. A flow diagram of this process is presented in Figure 1. The statistician who carried out the statistical analyses was blinded to the participants’ allocation.

### 2.4. Description of the Interventions

The experimental group received PNE plus usual care (physiotherapy). The control group only received usual care (physiotherapy). All the physiotherapists had more than twenty years of experience in the management of MSK pain disorders. The beliefs and attitudes of each physiotherapist and their clinical management with the biopsychosocial framework were assessed with the Spanish version of the Health Care Providers Pain and Impairment Relationship (HC-PAIRS) [23] scale. This tool ranges from 0 to 105, with greater scores reflecting a higher adherence to attitudes and beliefs regarding the notion that pain justifies disability and activity limitation. All the physiotherapists obtained similar scores (mean: 37.75; SD: ±6.18).

#### 2.4.1. Pain Neuroscience Education

PNE was delivered based on previous guidelines [24]. The physiotherapists applying this technique performed a total of 10 h of training in order to homogenize this intervention before the beginning of the study. One two-hour session of PNE was delivered during the first week of the trial. This session was conducted in small groups (5 participants to 5 companions) to maintain an appropriate dynamic. Audio-visual material using oral explanation, summaries, images, metaphors and diagrams via computers, as well as the use of written educational material, was delivered to each participant as reinforcement. The content of the educational session is described in Appendix A. A family member or friend accompanied the participants during the PNE session. This was to help improve the participant’s fidelity through sharing and confirming the new concepts in the participant’s direct environment. Furthermore, the recognition of the pain experience and the understanding of all the involved factors in the pain process were discussed during the educational session [25]. Each physiotherapist presented additional written information on the same day on which the educational session was provided. Participants were advised to read this information at least twice before the post-intervention assessment and three times more before the four-month follow-up assessment. Finally, physiotherapists discussed the concepts learned by the participants during usual care sessions to reinforce their knowledge about pain neurophysiology.

#### 2.4.2. Usual Care Intervention (Physiotherapy)

Usual care was provided for both groups over four weeks. It was based on manual therapy due to its potential analgesic effect through pain inhibitory mechanisms [26] and an exercise program that followed a standardized protocol based on a thorough prior dominant pain mechanism (nociceptive or central sensitization) [27,28,29,30].

Reminders were provided to all participants in order to guarantee the completion of the patient-reported questionnaires.

### 2.5. Outcome Measures

All the outcome measures were assessed at the baseline (t0), four weeks (t1) and four months (t2) after the beginning of the interventions.

#### 2.5.1. Primary Outcome

Self-efficacy beliefs were assessed using the Spanish version of the Chronic Pain Self-Efficacy Scale (CPSS) [31]. This tool is composed of 19 items in a 10-point Likert format, which ranges from 0 to 190 points, where the patients indicate the degree to which they consider themselves capable of performing certain activities or managing their pain, emotional problems, or other symptoms associated with chronic pain. High scores indicate greater self-efficacy beliefs. This questionnaire has shown a suitable level of internal consistency (Cronbach’s α coefficient = 0.91) among chronic pain patients [31]. Additionally, it has shown test-retest correlation coefficients of 0.68 for CPSS symptoms, 0.85 for CPSS physical, 0.46 for CPSS pain and 0.75 for CPSS total score, respectively.

#### 2.5.2. Secondary Outcomes

Pain intensity and pain interference were assessed using the Spanish version of the Graded Chronic Pain Scale (GCPS) [32]. This tool is comprised of 8 items, seven of them in the 10-point Likert format, with ranges from 0 to 70 points, where the participants indicate their pain intensity and pain interference. High scores indicate a higher perception of pain intensity and pain interference. This scale has demonstrated high internal consistency (Cronbach’s α = 0.87) and satisfactory test-retest reliability (intraclass correlation coefficient = 0.81) for chronic pain [32]. The minimum detectable change for a 95% confidence interval for this scale is 17.7 points [32].

Participants’ analgesics consumption was assessed by checking the digital medical history of each participant to register the amount (daily intake) and group (non-opioid, minor opioid, major opioid) prescribed by their physician, in order to continue the prescription along with the study and to evaluate possible changes in medication. Every change in pain intensity that could affect the patient´s medication was recorded, monitored and reported by the physiotherapists to the participant´s physician until solved.

The overall knowledge of pain neurophysiology of each participant was assessed using the revised Neurophysiology of Pain Questionnaire (NPQ) [33]. This tool is a 12-item version of the original NPQ. Each item required a response of “true”, “false” or “undecided”, and each item was scored, with one point awarded for each correct response and zero points for each incorrect or undecided response. High scores indicated a greater knowledge of pain neurophysiology. The original NPQ reported a reasonable internal consistency (person separation index = 0.84) and good test-retest reliability (intraclass correlation coefficient in pre-education = 0.97; and post-education = 0.98) [33]. The revised NPQ was collected two times during the pain neuroscience education session (pre-education and post-education). An adapted version specifically designed for the targeted population was created according to the existing guidelines [34].

Age, gender, height (cm), weight (kg), educational level (university, high school, secondary, elementary, no studies), employment situation (working, unemployed, sick leave, pensioner, homemaker) and pain duration (more than 3, 6, or 12 months) were also collected. Comorbidity was assessed using an adapted version of the Charlson Comorbidity Index [35].

### 2.6. Sample Size Calculation

To our knowledge, there have been no previous results on the use of the CPSS Spanish version in a similar study. Thus, an evaluation of 41 participants per group was needed for a 95% confidence level, a power of 95%, a ratio between samples (experimental: control) of 1:1, a one-tail consideration (in favor of the experimental group) [19] and an expected mean difference in CPSS total score of 10 points between groups after the intervention. This sample was expanded by 20% to minimize possible losses at follow-up, at one month and four months, with the final sample being 49 participants per group (a total of 98).

### 2.7. Statistical Analysis

A per-protocol analysis was conducted. Descriptive analyses using measures of central tendency, dispersion and position for quantitative variables, and frequency distribution for qualitative variables, were used. Differences between groups at the baseline were evaluated using the chi-square test (or Fisher’s exact test in the case of expected frequencies of 5) for qualitative variables and Student’s t-test for quantitative variables. Differences between groups for time-dependent outcome variables (t1 and t2) were analyzed using the generalized linear model for repeated measures, with a 95% confidence interval. In a secondary analysis, we investigated whether the knowledge acquired by participants in the intervention group (PNE plus usual care) about pain neurophysiology after treatment did/did not influence any of the outcome measures. This was assessed by exploring the differences in values of the revised NPQ for primary and secondary outcome measures at t1 (4-weeks) and t2 (4-months) time-points, in two subgroups. These subgroups were built based on the median score (8 points) attained in the revised NPQ. Then, the Mann–Whitney U test was used for quantitative variables and the Chi-square test for qualitative variables. In all the analyses, the level of statistical significance was established as *p* < 0.05. Effect sizes were reported using *d*-Cohen, with *d* = 0.2 being considered a “small” effect size, 0.5 a “medium” effect size and 0.8 a “large” effect size. An analysis of covariance (ANCOVA) was performed to address the potential effect of any participant´s characteristics on the primary and secondary outcomes, from the baseline to the four-month follow-up. The statistical analyses were conducted with SPSS statistical software (IBM Co, Armonk, NY, USA) for Windows, v.23.0.

## 3. Results

The recruitment included a total of 131 participants, with six participants declining to participate. A total of 125 participants were enrolled in the experimental (*n* = 67) and the control group (*n* = 58) and completed the baseline assessment. However, 16 participants did not conclude the study at the four-week follow-up, and nine participants did not conclude the study at the four-month follow-up. Ultimately, 100 participants completed the study (*n* = 49 experimental group; *n* = 51 control group) (Figure 1).

### 3.1. Demographic and Clinical Characteristics of the Sample

There were no statistically significant differences in demographic and clinical characteristics between the experimental and the control groups, except for pain duration. “Other predominantly MSK pain” was the largest group (71%), with the shoulder being the most affected region (Table 1).

### 3.2. PNE Plus Usual Care (Experimental Group) Versus Usual Care Alone (Control Group)

PNE plus usual care was more effective than usual care alone to enhance all the dimensions of self-efficacy beliefs across all the follow-up points (Table 2 and Table 3). PNE plus usual care was also more effective than usual care alone to reduce pain intensity and pain interference across all the follow-up points (Table 2 and Table 3).

### 3.3. PNE Plus Usual Care (Experimental Group) Versus Usual Care Alone (Control Group), Adjusted for Pain Duration

As the control and intervention groups were not matched for pain duration (Table 1), an ANCOVA was conducted, including pain duration (transformed in the dichotomic outcome: more than twelve months; less than twelve months) as a covariate. The statistically significant difference in the effectiveness of PNE plus usual care versus usual care alone at increasing self-efficacy beliefs and reducing pain intensity and pain interference remained after controlling for pain duration (Table 4 and Appendix B). No differences between groups were observed in terms of analgesics consumption (*p* = 0.07).

Finally, there were no statistically significant differences between groups regarding analgesics consumption at four-week and four-month follow-ups (*p* = 0.14). Additionally, no differences were detected in terms of participants’ changes from one analgesic therapeutic group to another (non-opioid, minor opioid, major opioid) when the experimental and control groups were compared (Table 5).

### 3.4. Secondary Analysis: Did Knowledge about Pain Neurophysiology Acquired after Intervention (Experimental Group) Influence the Effects Observed in the Different Outcomes?

The knowledge acquired by participants in the intervention group about pain neurophysiology did not modify the effects of PNE in any of the outcomes measured at the four-week (CPSS, *p* = 0.19; GCPS pain, *p* = 0.55; GCPS pain interference, *p* = 0.89; analgesics consumption, *p* = 0.79) and four-month (CPSS, *p* = 0.72; GCPS pain, *p* = 0.87; GCPS pain interference, *p* = 0.99; analgesics consumption, *p* = 0.80) follow-ups.

## 4. Discussion

This non-randomized trial evaluated whether the application of PNE plus usual care was more effective than usual care alone to improve self-efficacy beliefs, assessed with the CPSS in people with chronic MSK pain. We found statistically significant increases in favor of the experimental group when compared to the control group at both four-week and four-month follow-ups. Secondly, pain intensity and pain interference assessed with the GCPS also decreased with statistical significance in favor of the experimental group when compared to the control group at all the follow-ups. Thirdly, these results remained stable after controlling for pain duration. Fourthly, no differences were found between groups in terms of analgesics consumption. Finally, the knowledge acquired by participants in the intervention group about pain neurophysiology did not modify the effects of PNE in any of the outcome measures.

To our knowledge, this is the first trial analyzing the effectiveness of PNE plus usual care and using self-efficacy beliefs as a primary outcome in participants with chronic MSK pain. Contrary to our findings, a recent systematic review with meta-analyses [14] found that self-management interventions including educational strategies did not significantly improve pain self-efficacy when compared to controls after 0–3 months and 4–6 months. This discrepancy can be explained in three different ways. Firstly, none of the included trials in this systematic review applied PNE as an educational intervention. Secondly, the outcome measure in the previous systematic review was pain self-efficacy, while this trial assessed further dimensions of self-efficacy beliefs rather than pain self-efficacy alone. Thirdly, only randomized clinical trials were included in the aforementioned systematic review, which is not the case for our study (non-randomized trial design). The size of the effect on self-efficacy beliefs was medium at the 4-week and 4-month follow-ups. As far as we know, a minimal clinical detectable change has not been determined for CPSS; thus, we cannot know whether the findings of this study were meaningful for the participants. However, we believe that these results are novel in the field and should be considered. An increase in self-efficacy beliefs is one of the key points in reducing pain, disability and depressive symptoms [4,5]. Furthermore, they facilitate the engagement of valued activities [8] and increase the effects produced by cognitive behavioral interventions [9]. Participants in the PNE group could reconceptualize their pain through a better understanding of it and by recognizing how threatened they were feeling because of it. Nevertheless, this needs to be evaluated and corroborated with future randomized controlled trials.

Additionally, the results considering the reduction in pain intensity and pain interference after applying PNE are in consonance with the latest evidence. A systematic review [19] found improvements in pain intensity at short term (5.91 mm greater on the 100-mm in Visual Analog Scale -VAS, than control) and medium term (6.27 mm greater on the 100-mm VAS than control) after conducting several meta-analyses of nine trials and seven trials, respectively. The same systematic review showed a decrease in disability (pain interference), with a mean disability reduction in PNE of 4.09 out of 100 greater than controls at short term and 8.14 out of 100 greater at medium term. However, comparisons with our results are difficult due to the heterogeneity in the methods used to assess pain intensity and pain interference. According to the NICE guidelines for back and radicular pain, which sets the minimal clinically important change in clinical outcomes at 10%, the reported changes in the previous systematic review in pain intensity and disability are likely to be of little clinical benefit. In the same manner, our results did not surpass (10.2 points at 4-week follow-up and 12.8 points at 4-month follow-up) the minimal detectable change for the Spanish version of the GCPS (17.7 points) [32]. Even though we obtained statistically significant changes, with medium effect sizes for pain intensity and pain interference after applying PNE, it is plausible that greater changes in pain can occur in the long term so long as the pain is reconceptualized and that improvements in self-efficacy beliefs can remain. This should be corroborated in trials with longer follow-ups.

On the other hand, no differences between groups were found in terms of analgesics consumption. Previous evidence has shown inconsistencies regarding the effectiveness of PNE for the modification of the levels of analgesics consumption in people with MSK pain [36,37]. In this sense, further trials testing this issue should be conducted to establish further robust conclusions about this point. Finally, the knowledge acquired by participants in the intervention group about pain neurophysiology did not modify the effects of PNE in any of the outcome measures. This finding may suggest that other relevant factors associated with the development of educational pain sessions, such as exploring the patient’s context, making sense of the patient’s context and tailoring education, may be involved [38]. However, this finding should be replicated for randomized controlled trials in different samples in order to facilitate firm conclusions.

### 4.1. Strengths and Limitations

There are some strengths to this study. Self-efficacy beliefs were evaluated as a primary outcome. Additionally, the mediating role of knowledge about pain neurophysiology was analyzed. However, some limitations should be recognized. Firstly, the present trial was a non-randomized study. This type of design may lead to systematic bias from confounding by clinical indication and, without random allocation, treatment effects are possibly affected not only by systematic bias but also by increased uncertainty [39]. Future large randomized controlled trials evaluating the effectiveness of PNE for the modification of self-efficacy beliefs may reduce these biases. Secondly, all the outcome measures were assessed using self-report tools. In this sense, the presence of social desirability bias should be considered. Future studies that aim at evaluating outcome measures with objective measurements, e.g., accelerometers, are needed. Thirdly, the blinding of the treatment providers was impossible due to the nature of the interventions. In this context, future trials are needed that use a similar control group—for example, another education pain intervention may facilitate the blinding of health providers. Finally, all the therapists presented similar attitudes and beliefs (pain does not justify disability and activity limitation), which is experienced in the biopsychosocial framework. Thus, this may have introduced bias in the control group, as therapists could unconsciously provide information about pain neuroscience to the participants.

### 4.2. Clinical Implications

Clinicians are encouraged to use PNE as an optional educational strategy to improve self-efficacy beliefs in patients with chronic MSK pain. Physiotherapists may help patients to build self-efficacy beliefs, foster the patients’ ability to attain a goal [13], provide reassurance and guide problem-solving in order to help patients overcome barriers [40]. This may encourage patients to be more open to active interventions such as exercise [19]. Despite their potential benefits/advantages, PNE presents some a priori disadvantages that need to be considered: (I) it implies time-consuming planning (e.g., elaboration of specific materials for the educational session); (II) it requires special rooms in which the treatment can be carried out; (III) it is necessary to develop specific skills relating to different groups dynamics, i.e., to educate people with different ideas and backgrounds about pain neurophysiology. However, the findings should be interpreted with caution as we do not know whether the differences found in CPSS between groups were clinically significant for patients.

## 5. Conclusions

Despite its limitations, the present non-randomized trial found that the application of PNE plus usual care is more beneficial than usual care alone to enhance self-efficacy beliefs and reduce pain intensity and pain interference in people with chronic MSK pain. These improvements were maintained after controlling for pain duration. On the other hand, no differences between groups were found regarding analgesics consumption. Finally, the knowledge acquired by participants in the intervention group about pain neurophysiology did not modify the effects of PNE in any of the outcome measures. These results are promising but need to be confirmed in a larger, methodological sound trial.

## Figures and Tables

**Table 1:** Demographic and clinical characteristics of the total sample (*n* = 100).

Variable		Experimental (*n* = 49)	Control (*n* = 51)
Gender (%)	Male	12.2%	19.6%
	Female	87.8%	80.4%
Age, years (mean ± SD)		47.2 (±8.5)	48.6 (±9.5)
Body mass index (mean ± SD)		26.1(±4.4)	25.8 (±4.8)
Educational level (%)	University	12.2	5.9
	High school	22.4	11.8
	Secondary	32.7	39.2
	Elementary	32.7	43.1
	No formal studies	0.0	0.0
Employment situation (%)	Unemployed	24.5	17.6
	Sick leave	0.0	5.9
	Pensioner	4.1	3.9
	Housework	10.2	11.8
	Working	61.2	60.8
Pain duration (%) *	>3 months	2.0	7.8
	>6 months	6.1	19.6
	>12 months	91.8	72.5
Charlson comorbidity index (%)	No comorbidity	87.8	94.1
	Low comorbidity	8.2	3.9
	High comorbidity	4.1	2.0
ACTTION–AAPT (%)	Osteoarthritis	0.0	0.0
	Other arthritis (e.g., rheumatoid arthritis, gout, connective tissue diseases)	0.0	0.0
	MSK lower back pain	32.7	25.5
	Myofascial pain, chronic widespread pain and fibromyalgia	0.0	0.0
	Other predominantly MSK pain	67.3	74.5
Other MSK pain conditions (%)	Shoulder	40.8	37.3
	Lumbar spine	32.7	25.5
	Neck	22.4	13.7
	Knee	4.1	9.8
	Hip	0.0	13.7
CPSS (mean ± SD)	Symptoms	53.3 (±14.7)	48.5 (±16.9)
	Physical	46.9 (±11.0)	44.6 (±14.9)
	Pain	31.5 (±12.0)	28.1 (±13.1)
GCPS (mean ± SD)	Pain	19.4 (±5.2)	20.8 (±5.0)
	Pain interference	18.7 (±10.2)	21.9 (±11.1)
Analgesic consumption (mean ± SD)		0.7 (±1.2)	0.7 (±1.1)
Therapeutic group of analgesic medication (%)	None	67.3	58.8
	Non-opioids	16.3	35.3
	Minor opioids	16.3	3.9
	Major opioids	0.0	2.0

GCPS = Graded Chronic Pain Scale; CPSS = Chronic Pain Self-Efficacy Scale; (*p* < 0.05); * there are statistically differences between groups.

**Table 2:** Mean values (SD) and mean differences between groups regarding self-efficacy beliefs, pain intensity and pain interference at 4-week follow-up (post-treatment).

Four-Week Follow-up	Experimental (*n* = 49) Mean (SD)	Control (*n* = 51) Mean (SD)	Group Difference, Mean (95% CI)	*p*-Value	*d*-Cohen
CPSS					
Symptoms	61.5 (16.5)	49.9 (18.2)	11.5 (4.6; 18.5)	0.001 *	0.67
Physical	53.8 (9.0)	44.4 (16.0)	9.3 (4.1; 14.5)	0.001 *	0.69
Pain	37.7 (10.0)	29.7 (13.1)	7.9 (3.3; 12.6)	0.001 *	0.69
Total score	153.0 (30.6)	124.1 (44.1)	28.9 (13.8; 44.1)	<0.001 *	0.76
GCPS					
Pain	13.7 (5.0)	16.8 (5.9)	−3.16 (−5.3; −0.9)	0.005 *	0.57
Pain interference	11.1 (8.9)	18.1 (11.3)	−7.0 (−11.0; −2.9)	0.001 *	0.69
Total score	24.8 (13.0)	35.0 (15.3)	−10.1 (−15.8; −4.5)	0.001 *	0.72

GCPS = Graded Chronic Pain Scale; CPSS = Chronic Pain Self-Efficacy Scale; * differences statistically significant (*p* < 0.05).

**Table 3:** Mean values (SD) and mean differences between groups regarding self-efficacy beliefs, pain intensity and pain interference at 4-month follow-up.

Four-Month Follow-up	Experimental (*n* = 49) Mean (SD)	Control (*n* = 51) Mean (SD)	Group Difference, Mean (95% CI)	*p*-Value	*d*-Cohen
CPSS					
Symptoms	65.4 (14.8)	55.3 (17.9)	10.1 (3.5; 16.6)	0.003 *	0.61
Physical	52.8 (10.7)	45.8 (15.1)	6.9 (1.7; 12.2)	0.009 *	0.99
Pain	40.3 (10.3)	29.5 (13.8)	10.7 (5.9; 15.6)	<0.001 *	0.88
Total score	158.6 (33.1)	130.8 (42.8)	27.8 (12.6; 43.1)	<0.001 *	0.73
GCPS					
Pain	11.1 (6.9)	15.4 (7.1)	−4.3 (−7.1; −1.5)	0.003 *	0.61
Pain interference	6.0 (8.9)	14.4 (12.2)	−8.4 (−12.7; −4.1)	<0.001 *	0.79
Total score	17.1 (14.5)	29.9 (18.5)	−12.7 (−19.4; −6.1)	<0.001 *	0.77

GCPS = Graded Chronic Pain Scale; CPSS = Chronic Pain Self-Efficacy Scale; * differences statistically significant (*p* < 0.05).

**Table 4:** Estimated marginal mean values and mean differences between groups regarding self-efficacy beliefs, pain intensity and pain interference (ANCOVA).

	Experimental (*n* = 49) Estimated Marginal Mean	Control (*n* = 51) Estimated Marginal Mean	Group Difference, Mean (95% CI)	*p*-Value
CPSS				
Symptoms	60.8 (56.8; 64.8)	50.5 (46.6; 54.4)	10.3 (4.6; 15.9)	<0.001 *
Physical	51.9 (48.5; 55.3)	44.2 (40.9; 47.6)	7.6 (2.7; 12.5)	0.002 *
Pain	37.1 (34.1; 40.2)	28.5 (25.5; 31.5)	8.6 (4.3; 12.9)	<0.001 *
Total score	149.9 (140.4; 159.5)	123.3 (113.9; 132.7)	26.6 (13.0; 40.2)	<0.001 *
GCPS				
Pain	14.5 (13.2; 15.9)	17.6 (16.3; 18.9)	−3.0 (−4.9; −1.1)	0.002 *
Pain interference	11.6 (9.0; 14.2)	18.5 (15.9; 21.0)	−6.8 (−10.5; −3.2)	<0.001 *
Total score	26.2 (22.5; 29.8)	36.1 (32.5; 39.7)	−9.9 (−15.1; −4.7)	<0.001 *

* *p* < 0.05; GCPS = Graded Chronic Pain Scale; CPSS = Chronic Pain Self-Efficacy Scale. Pain duration was included as a covariate.

**Table 5:** Changes in the therapeutic group of analgesic medication.

	Experimental (*n* = 49)			Control (*n* = 51)			
	Increase %	No Change %	Decrease %	Increase %	No Change %	Decrease %	*p*-Value
**Four-Week Follow-Up**	0.0	77.6	22.4	2.0	88.2	9.8	0.089 *
**Four-Month Follow-Up**	0.0	75.5	24.5	5.9	78.4	15.7	0.270 *

* *p* < 0.05.

**Figure A1:**
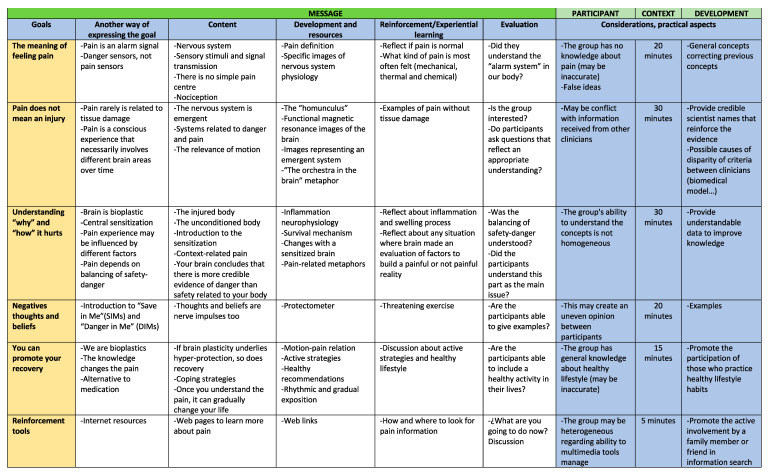
Curriculum for 120 min of Pain Neuroscience Education, in a group session, for patients with chronic musculoskeletal pain, based on a previous guideline [41].

**Figure A2:**
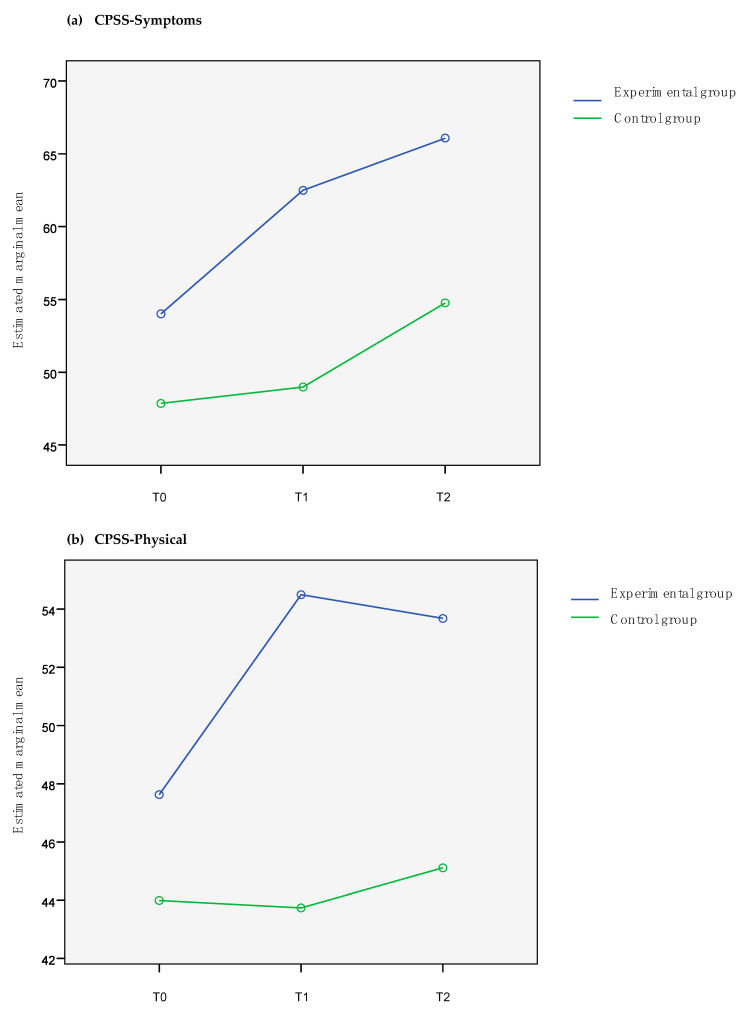

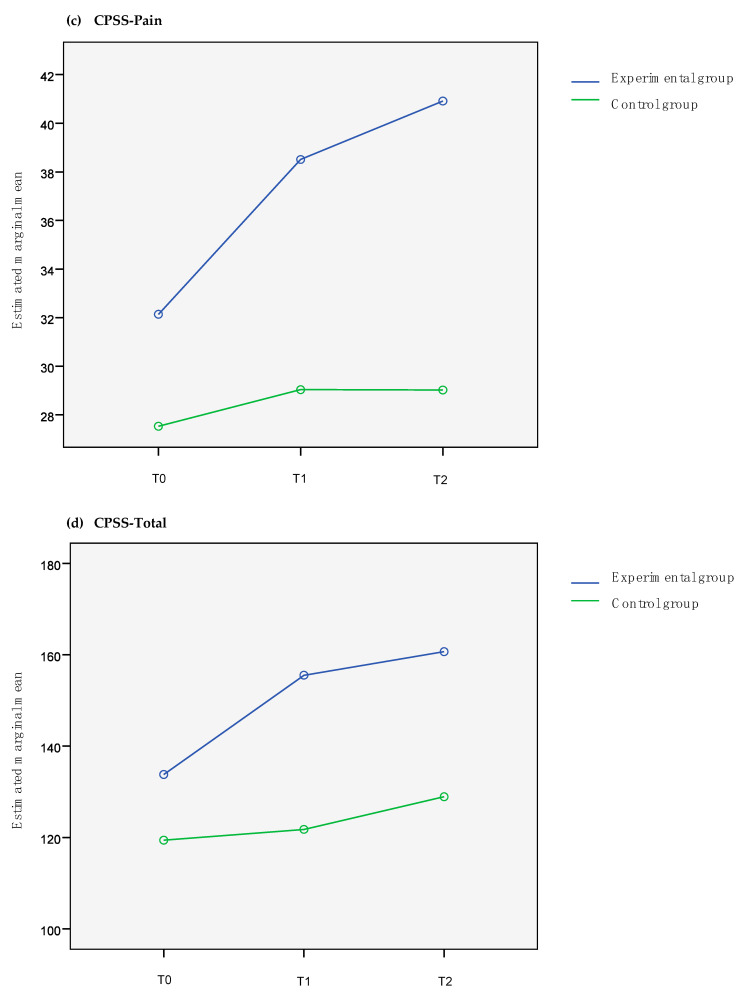

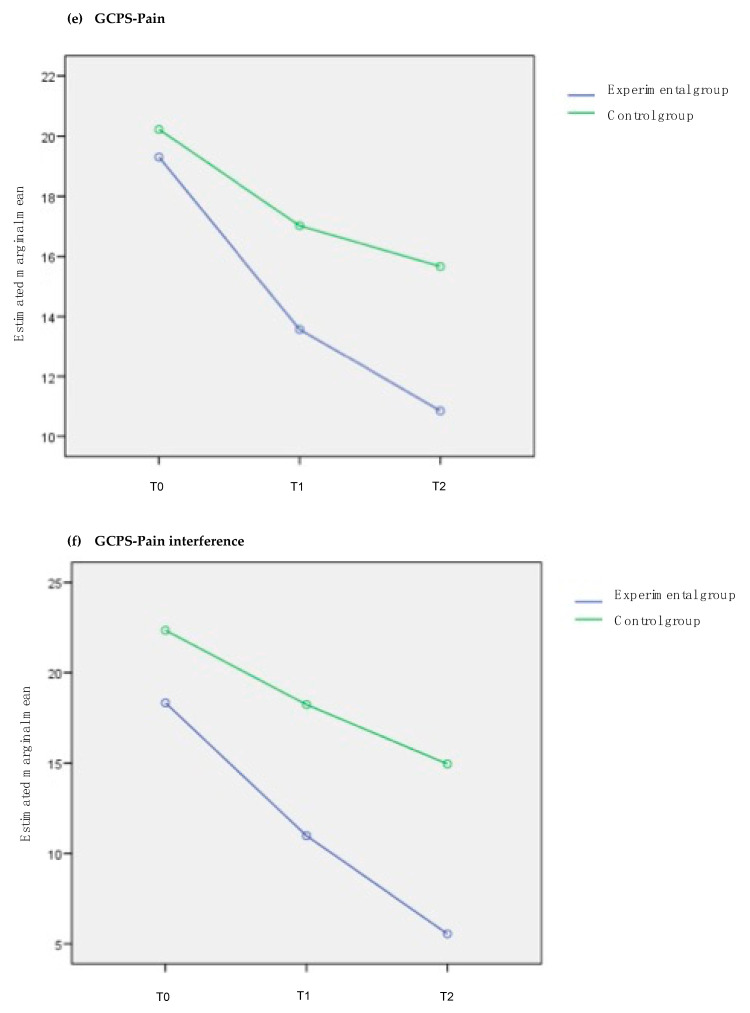

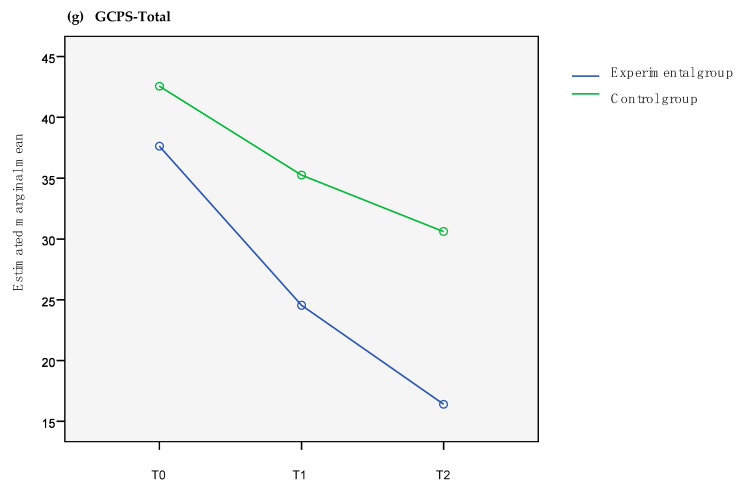
Estimated marginal mean values between groups regarding self-efficacy beliefs, pain intensity and pain interference (ANCOVA). (**A**) Symptoms domain of Chronic Pain Self-Efficacy Scale (CPSS); (**B**) Physical domain of CPSS; (**C**) Pain domain of CPSS; (**D**) CPSS total score; (**E**) Pain intensity domain of Graded Chronic Pain Scale (GCPS); (**F**) Pain interference domain of GCPS; (**G**) GCPS total score.

## Appendix B

Estimated marginal mean values between groups regarding self-efficacy beliefs, pain intensity and pain interference (ANCOVA).
